# Deep learning for predicting major pathological response to neoadjuvant chemoimmunotherapy in non-small cell lung cancer: A multicentre study

**DOI:** 10.1016/j.ebiom.2022.104364

**Published:** 2022-11-14

**Authors:** Yunlang She, Bingxi He, Fang Wang, Yifan Zhong, Tingting Wang, Zhenchuan Liu, Minglei Yang, Bentong Yu, Jiajun Deng, Xiwen Sun, Chunyan Wu, Likun Hou, Yuming Zhu, Yang Yang, Hongjie Hu, Di Dong, Chang Chen, Jie Tian

**Affiliations:** a Department of Thoracic Surgery, Shanghai Pulmonary Hospital, School of Medicine, Tongji University, Shanghai, China; b Beijing Advanced Innovation Center for Big Data-Based Precision Medicine, School of Engineering Medicine, Beihang University, Beijing, China; c Key Laboratory of Big Data-Based Precision Medicine, Beihang University, Ministry of Industry and Information Technology, Beijing, China; d CAS Key Laboratory of Molecular Imaging, The State Key Laboratory of Management and Control for Complex Systems, Institute of Automation, Chinese Academy of Sciences, Beijing, China; e Department of Radiology, Sir Run Run Shaw Hospital, Zhejiang University School of Medicine, Hangzhou, China; f Department of Radiology, Shanghai Pulmonary Hospital, School of Medicine, Tongji University, Shanghai, China; g Department of Thoracic Surgery, Shanghai Tongji Hospital, School of Medicine, Tongji University, Shanghai, China; h Department of Thoracic Surgery, Hwa Mei Hospital, Chinese Academy of Sciences, Zhejiang, China; i Department of Thoracic Surgery, The First Affiliated Hospital of Nanchang University, Nanchang, China; j Department of Pathology, Shanghai Pulmonary Hospital, School of Medicine, Tongji University, Shanghai, China; k School of Artificial Intelligence, University of Chinese Academy of Sciences, Beijing, China; l Engineering Research Center of Molecular and Neuro Imaging of Ministry of Education, School of Life Science and Technology, Xidian University, Xi'an, China

**Keywords:** Deep learning, Neoadjuvant chemoimmunotherapy, Major pathological response, Non-small cell lung cancer

## Abstract

**Background:**

This study, based on multicentre cohorts, aims to utilize computed tomography (CT) images to construct a deep learning model for predicting major pathological response (MPR) to neoadjuvant chemoimmunotherapy in non-small cell lung cancer (NSCLC) and further explore the biological basis under its prediction.

**Methods:**

274 patients undergoing curative surgery after neoadjuvant chemoimmunotherapy for NSCLC at 4 centres from January 2019 to December 2021 were included and divided into a training cohort, an internal validation cohort, and an external validation cohort. ShuffleNetV2x05-based features of the primary tumour on the CT scans within the 2 weeks preceding neoadjuvant administration were employed to develop a deep learning score for distinguishing MPR and non-MPR. To reveal the underlying biological basis of the deep learning score, a genetic analysis was conducted based on 25 patients with RNA-sequencing data.

**Findings:**

MPR was achieved in 54.0% (n = 148) patients. The area under the curve (AUC) of the deep learning score to predict MPR was 0.73 (95% confidence interval [CI]: 0.58–0.86) and 0.72 (95% CI: 0.58–0.85) in the internal validation and external validation cohorts, respectively. After integrating the clinical characteristic into the deep learning score, the combined model achieved satisfactory performance in the internal validation (AUC: 0.77, 95% CI: 0.64–0.89) and external validation cohorts (AUC: 0.75, 95% CI: 0.62–0.87). In the biological basis exploration for the deep learning score, a high deep learning score was associated with the downregulation of pathways mediating tumour proliferation and the promotion of antitumour immune cell infiltration in the microenvironment.

**Interpretation:**

The proposed deep learning model could effectively predict MPR in NSCLC patients treated with neoadjuvant chemoimmunotherapy.

**Funding:**

This study was supported by National Key Research and Development Program of China, China (2017YFA0205200); National Natural Science Foundation of China, China (91959126, 82022036, 91959130, 81971776, 81771924, 6202790004, 81930053, 9195910169, 62176013, 8210071009); Beijing Natural Science Foundation, China (L182061); Strategic Priority Research Program of Chinese Academy of Sciences, China (XDB38040200); Chinese Academy of Sciences, China (GJJSTD20170004, QYZDJ-SSW-JSC005); Shanghai Hospital Development Center, China (SHDC2020CR3047B); and Science and Technology Commission of Shanghai Municipality, China (21YF1438200).

**Research in context**

**Evidence before this study**

We searched PubMed (https://pubmed.ncbi.nlm.nih.gov/) for studies assessing the predictive value of radiomics for neoadjuvant therapy response in lung cancer. We found several studies (PMID: PMC5318226; PMID: PMC4930885) attempted to use radiomics phenotypes to predict pathological response to neoadjuvant chemotherapy in NSCLC. In 2016, Coroller et al. extracted the radiomics features of primary tumour based 127 NSCLC patients treated by neoadjuvant chemotherapy, demonstrating that the Wavelet HLL mean was the only significantly predictive feature (AUC = 0.63, P-value = 0.01) for a pathological complete response. In 2017, Coroller et al. further investigated the value of radiomic data of the primary tumour and lymph nodes in predicting pathological response after neoadjuvant chemotherapy. They established a predictive model based on 85 patients, achieving an AUC of 0.73. Yet despite these efforts, existing publications only limited in population of neoadjuvant chemotherapy; thus, the predictive value of radiomics for pathological response to neoadjuvant chemoimmunotherapy which serves as a promising treatment for NSCLC, remains uncertain. In addition, the previously cited studies were based on a single-centre design whereas the predictive value of radiomics requires robust external validation. Finally, deep learning is a new branch of radiomics, and its role in predicting response to neoadjuvant chemoimmunotherapy warrants further evaluation.

**Added value of this study**

The current multicentre study constructed a CT-based deep learning model to predict MPR probability in NSCLC after neoadjuvant chemoimmunotherapy. By integrating the deep learning score and clinical score, the combined model achieved AUCs of 0.77 and 0.75 in the internal and external validation cohorts, respectively. Furthermore, we investigated the biological basis of the deep learning prediction, proving that a high deep learning score was associated with the downregulation of pathways for mediating tumour proliferation and the promotion of antitumour immune cell infiltration in the microenvironment.

**Implications of all the available evidence**

The proposed deep learning model can effectively predict MPR in NSCLC patients treated with neoadjuvant chemoimmunotherapy, which would promote personalized medicine for NSCLC patients.

## Introduction

For early and locally advanced non-small cell lung cancer (NSCLC), a surgical resection continues to the mainstay of curative-intent therapeutic strategies.^1^ However, even with a radical resection, disease control remains dismal, with postsurgical relapse occurring in 30%–55% of patients.^2^ Moreover, the addition of neoadjuvant or adjuvant chemotherapy confers limited benefits in preventing recurrence, leading to an improvement of only 5% in overall survival,^3^ which promotes calls for therapeutic innovation in resectable NSCLC. In the last several years, immunotherapy comprising immune checkpoint inhibitors (ICIs) has demonstrated tremendous antitumour efficacy and dramatically shifted the treatment paradigms of advanced NSCLC,^4^ providing a credible rationale for the implementation of immunotherapy in the neoadjuvant setting for resectable NSCLC.

The feasibility and efficacy of neoadjuvant immunotherapy have been investigated by multiple studies,^5^, ^6^, ^7^, ^8^ indicating that neoadjuvant immunotherapy could reduce tumour burden prior to surgery, potentially increasing the chance of radical resection while controlling micrometastases in early phases, thereby mitigating the recurrence risk. In this context, neoadjuvant immunotherapy has shed new light on the potential therapeutic breakthroughs of resectable NSCLC. Nevertheless, a significant percentage of patients could not achieve major pathological response (MPR) through the neoadjuvant immunotherapy.^9^ Recently, preliminary results of the NADIM study revealed that MRP was associated with significantly improved 1-year progression free survival (88.4% versus 57.1%, P = 0.01) in NSCLC patients undergoing neoadjuvant chemoimmunotherapy,^7^ further emphasizing the importance of MPR in evaluating neoadjuvant immunotherapeutic efficacy for NSCLC. As such, developing a robust biomarker for MPR to neoadjuvant chemoimmunotherapy in resectable NSCLC is of paramount importance.

Deep learning, capable of quantifying the high-dimensional radiological phenotypes that cannot be captured by the human eye and directly developing targeted predictive models for various clinical scenarios,^10^ provided a noninvasive instrument for disease diagnosis,^11^ therapeutic decision,^12^ and prognosis evaluation.^13^ Previous publications have revealed that the deep learning models could effectively distinguish TMB and PD-L1 status, thereby screening out advanced NSCLC patients benefiting from chemoimmunotherapy.^14^^,^^15^ In addition, the deep learning imaging score could directly predict the prognosis of chemoimmunotherapy in advanced NSCLC,^16^ which implied the associations between deep learning features and chemoimmunotherapy response, and laid the theoretical foundation of the predictive value of deep learning in the neoadjuvant context. However, no evidence indicates feasibility of the deep learning technique in predicting the response of neoadjuvant chemoimmunotherapy for NSCLC. The current study, based on multicentre cohorts, purposes to utilize computed tomography (CT) images to construct and validate a deep learning model for predicting MPR in NSCLC patients treated by neoadjuvant ICIs combined with chemotherapy and further explore the biological basis under its prediction.

## Methods

### Ethics

The study was approved by the Ethics Committee (L20-333-1) of Shanghai Pulmonary Hospital, Ningbo Hwa Mei Hospital, The First Affiliated Hospital of Nanchang University and Sir Run Run Shaw Hospital. Informed consent was waived due to the retrospective nature of this study.

### Patients

Patients undergoing curative surgery after neoadjuvant chemoimmunotherapy for NSCLC at Shanghai Pulmonary Hospital, Ningbo Hwa Mei Hospital, The First Affiliated Hospital of Nanchang University and Sir Run Run Shaw Hospital from January 2019 to December 2021 were included. Patients were excluded when they met either of the following criteria: missing image data and pathological N3 disease. The baseline characteristics and chest CT images taking in the 2 weeks preceding neoadjuvant administration were retrospectively collected.

### Pretreatment evaluation and neoadjuvant administration

A full evaluation of tumour diagnosis and staging was conducted before neoadjuvant therapy and included a CT scan, abdominal ultrasound, magnetic resonance imaging of the cerebrum, positron emission tomography, and endobronchial ultrasound-guided transbronchial fine needle aspiration. The neoadjuvant chemoimmunotherapy regimens consisted of 2–4 cycles (3 weeks per cycle) of pembrolizumab (200 mg) or nivolumab (360 mg) combined with platinum-based chemotherapy. The physician in charge decided which immune-checkpoint inhibitor and treatment cycles to administer. Surgical resection with systematic lymph node dissection was planned at around 4 weeks after the completion of neoadjuvant therapy.

### Pathological evaluation

The pathological response was assessed according to the following criteria,^17^ as the percentages of viable tumour cells, necrosis and stroma were respectively determined. The MPR was defined as no more than 10% of a viable tumour in the primary tumour bed, and the circumstance in which no viable tumour was observed was defined as a pathologic complete response (pCR). All specimens were evaluated by two independent pathologists (L.K.H. and C.Y.W.) with more than 15 years of experience, disagreements were resolved by consensus after discussion.

### CT examination and tumour annotation

Chest CT scans were manufactured by Siemens (Somatom Definition AS+, Biograph64), Philips (Brilliance 40, iCT256, Ingenuity Flex, MX 16-slice), GE Medical System (Bright Speed), and United Imaging (uCT 510, uCT 760, uCT S-160). All images were reconstructed and then imported into 3D slicer (http://www.slicer.org) for annotation.

The region of interest (ROI) was annotated by a bounding box that included the entire tumour volume. Two radiologists (T.T.W. and Y.Y.) with 5 years of experience independently performed tumour annotations in the lung window setting (mean, −450 HU; width, 1500 HU), and interobserver disagreements were resolved by consulting a senior radiologist (X.W.S.) with more than 10 years of experience.

A key issue is that the original CT images have different voxel lengths. Thus, before feeding data into our network, we interpolated the original images to the same voxel spacing (1 mm × 1 mm × 1 mm). We also used Slope and Intercept in the dicom header information to standardize tumour images to HU values, and set cut-off value to prevent extreme values in images. We calculated the mean and variance of three-dimensional (3D) tumour images in the training cohort and normalized all images by Z-score. This normalization method, which conforms to the normal distribution, could facilitate the network learning. Finally, we generated new data for training by shifting the bounding box of several voxels in different directions to mitigate the intra- and inter-observer differences created by annotations.

### Model construction and validation

**Fig. 1:**
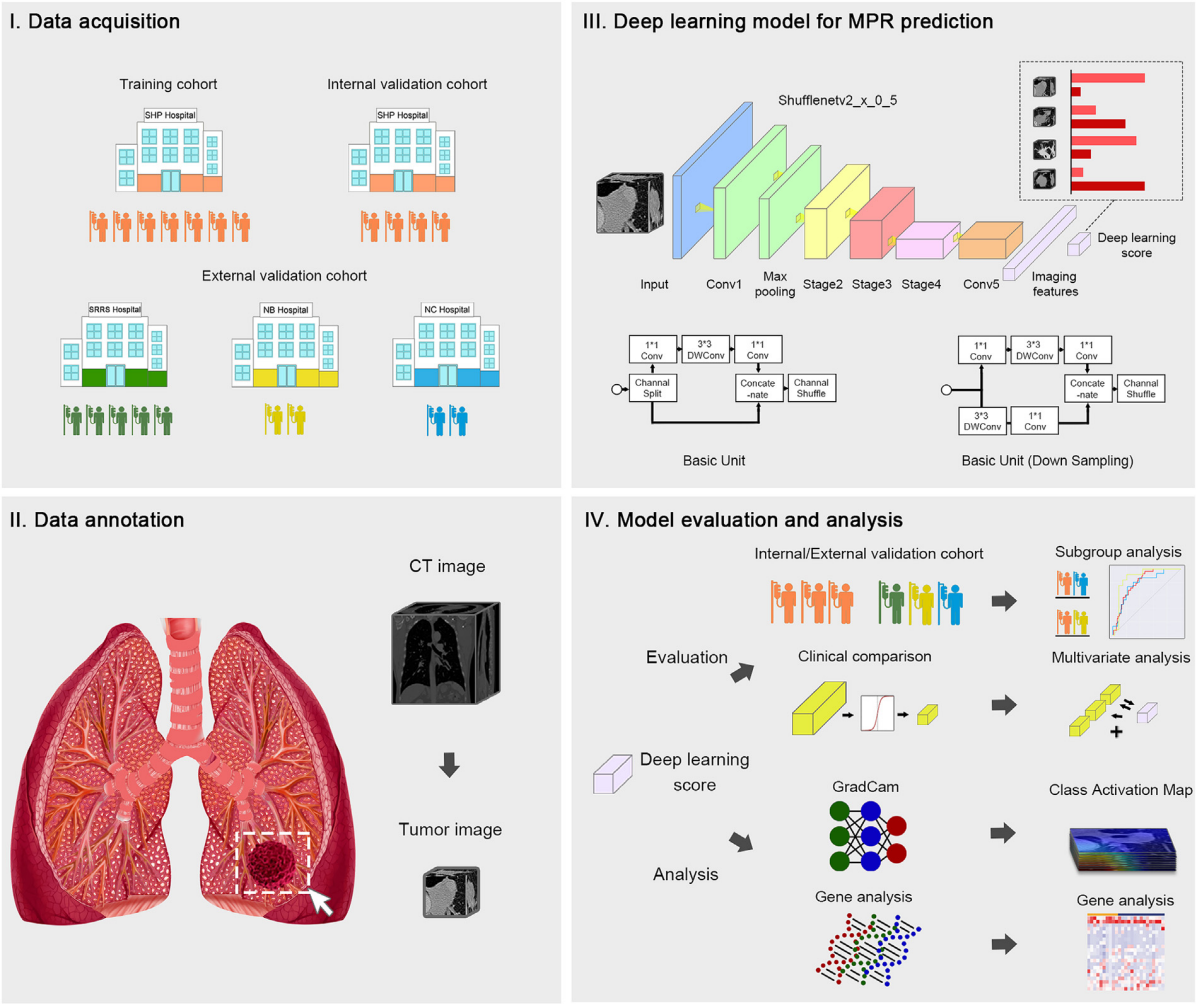
**Flowchart illustrates the study design**. (Ⅰ) A total of 274 patients acquired from four institutions are divided into three cohorts; (Ⅱ) The region of interest is annotated with a bounding box covering the lesion on CT images; (Ⅲ) The deep learning model is built using convolutional neural network algorithm; (IV) The predictive efficiency of the deep learning model is assessed in the internal/external validation cohorts, and the underlying predictive mechanism of the deep learning model is investigated with the visual analysis and genetic analysis.

We developed a deep learning model for predicting MPR to neoadjuvant chemoimmunotherapy via a convolutional neural network. Before constructing the model, we randomly stratified all patients into cohorts by the centres (Fig. 1). Patients in Shanghai Pulmonary Hospital were divided into a training cohort and an internal validation cohort at a ratio of seven to three, and 25 patients with genetic data were guaranteed to be included in the internal validation cohort. In addition, all patients at Ningbo Hwa Mei Hospital, The First Affiliated Hospital of Nanchang University and Sir Run Run Shaw Hospital were considered to be the external validation cohort.

The complete model contained a convolution module and a classification module, and was constructed in three main steps: 1) using a convolutional network to connect a linear classifier to obtain an effective feature extractor; 2) freezing the convolutional network to dock a fully connected network with 512 hidden layer nodes was used to further integrate high-dimensional features to obtain high-precision classifiers; and 3) unfreezing all parameters of the network for end-to-end training with a small learning rate. For the convolutional network, we intended to use 3D-ShuffleNetv2x05 for the feature extraction of images in the bounding box. The 3D-ShuffleNetv2x05 is the 3D version of the model, which was a mainstream lightweight neural network with the smallest number of parameters in ShuffleNetv2.^18^ The body of Shufflenetv2 contains five modules, which we named Conv1+maxpool, Stage 2, Stage 3, Stage 4, and Conv 5. Meanwhile, we also compared the results of 3D-Resnet18,^19^ 3D-densenet121^20^ and 3D-MobilenetV3^21^ as convolutional networks. For the fully connected network, we added a hidden layer with 512 nodes before the output layer to further fuse the features obtained by the convolutional network.

As the Softmax function is added to the output layer, we directly obtained the MPR probability by the model output. To assess the stability of the constructed model, we validated the deep learning model and compared it with a clinical model in the internal validation and external validation cohorts. Furthermore, we combined the deep learning and clinical models to generate a combined model to comprehensively evaluate the efficiency of the imaging marker.

### Biological basis exploration

To explore the underlying biological basis of the deep learning prediction, gene analyses were conducted among 25 patients with RNA-sequencing data in the internal validation cohort. Using the median value of the deep learning score, the 25 patients were divided into 10 patients with a high deep learning score and 15 patients with a low deep learning score. The R package limma was used to identify differential expression genes between two groups according to the criteria of log FC >2 and adjusted P value <0.05. Subsequently, Kyoto Encyclopedia of Genes and Genomes (KEGG) analyses were conducted using the R package clusterProfiler to identify the signalling pathways related to the deep learning score. Immune microenvironment analyses were performed via CibersortX (https://cibersortx.stanford.edu) to estimate the abundances of member cell types in a mixed cell population.

### Statistical analysis

The category and continuous baseline characteristics of patients were described using frequency (percentage) and mean ± standard deviation, which were compared using a Chi-square test and t test, respectively. The receiver operating characteristic curve (ROC) and area under the curve (AUC) were mainly used to evaluate the efficiency of the model. The Delong test was used to calculate the difference between the AUCs. In addition, we used the bootstrap method to calculate the confidence interval by repeating the sampling with replacement 500 times. All analyses were performed in R (version 3.5.2; http://www.R-project.org) and Python (version 3.6.7; http://www.python.org/). P values less than 0.05 were considered a significant difference. The Python and R packages are presented in the Supplement.

### Role of the funding source

The funder of the study played no role in the study design, data collection, data analysis, data interpretation, or writing of the report.

## Results

### Clinicopathological characteristics

The baseline information is summarized in Table 1. In total, 274 patients were included in this study, and divided into the training cohort, internal validation cohort and external validation cohort (142, 61, and 71 patients, respectively). R0 resections were achieved for all included patients. The mean age of the entire cohort was 62.1 years; 88.3% (n = 242) patients were male. There were 170 (62.0%) squamous cell carcinomas and 82 (29.9%) adenocarcinomas. With respect to pretreatment staging, most patients were evaluated as T2 (n = 109, 39.8%) and N2 (n = 173, 63.1%) stage, and stage III (n = 225, 82.1%) accounted for the largest proportion of the whole population. In the evaluation of the pathological response, most patients (n = 148, 54.0%) were evaluated as MPR, and pCR was achieved in 29.9% (n = 82) of patients. Compared to the internal cohort, an earlier pretreatment stage (T1 stage: 38.0% versus 16.4%, P < 0.001; stage I: 15.5% versus 0%, P < 0.001) and higher proportion of squamous cell carcinoma (77.5% versus 57.4%, P < 0.001) were observed patients in the external cohort had and the remaining characteristics were similar between two cohorts.

**Table 1:** Clinicopathological characteristics of all included patients.

Characteristics	Entire cohort (n = 274)	Internal cohort (n = 203)		External validation cohort (n = 71)	P value^a^
		Training cohort (n = 142)	Internal validation cohort (n = 61)		
Age, mean ± SD, years	62.1 ± 8.7	61.4 ± 9.3	61.8 ± 8.6	63.6 ± 7.3	0.38
<65	151 (55.1)	79 (55.6)	35 (57.4)	37 (52.1)	
≥65	123 (44.9)	63 (44.4)	26 (42.6)	34 (47.9)	
Gender					0.07
Female	32 (11.7)	21 (14.8)	8 (13.1)	3 (4.2)	
Male	242 (88.3)	121 (85.2)	53 (86.9)	68 (95.8)	
Smoking status					0.23
Never smoked	145 (52.9)	82 (57.7)	28 (45.9)	35 (49.3)	
Smoker	129 (47.1)	60 (42.3)	33 (54.1)	36 (50.7)	
Pretreatment T stage					0.11
T1	41 (15)	22 (15.5)	9 (14.8)	10 (14.1)	
T2	109 (39.8)	60 (42.3)	19 (31.1)	30 (42.3)	
T3	64 (23.4)	33 (23.2)	11 (18)	20 (28.2)	
T4	60 (21.9)	27 (19.0)	22 (36.1)	11 (15.5)	
Pretreatment N stage					<0.001
N0	52 (19)	15 (10.6)	10 (16.4)	27 (38)	
N1	49 (17.9)	29 (20.4)	6 (9.8)	14 (19.7)	
N2	173 (63.1)	98 (69)	45 (73.8)	30 (42.3)	
Pretreatment TNM stage					<0.001
I	13 (4.7)	2 (1.4)	0	11 (15.5)	
II	36 (13.1)	15 (10.6)	5 (8.2)	16 (22.5)	
III	225 (82.1)	125 (88)	56 (91.8)	44 (62)	
Surgical procedure					0.67
Lobectomy	223 (81.4)	115 (81)	51 (83.6)	57 (80.3)	
Bilobectomy	31 (11.3)	16 (11.3)	8 (13.1)	7 (9.9)	
Pneumonectomy	20 (7.3)	11 (7.7)	2 (3.3)	7 (9.9)	
Pathological N stage					0.88
N0	179 (65.3)	90 (63.4)	43 (70.5)	46 (64.8)	
N1	34 (12.4)	19 (13.4)	7 (11.5)	8 (11.3)	
N2	61 (22.3)	33 (23.2)	11 (18)	17 (23.9)	
N downstage in pretreatment N2 disease					0.58
N2 to N0	105 (60.7)	59 (60.2)	31 (68.9)	15 (50)	
N2 to N1	25 (14.5)	15 (15.3)	5 (11.1)	5 (16.7)	
N2	43 (24.9)	24 (24.5)	9 (20)	10 (33.3)	
Histology					0.02
SCC	170 (62.1)	80 (56.3)	35 (57.4)	55 (77.5)	
ADC	82 (29.9)	48 (33.8)	19 (31.1)	15 (21.1)	
Others	22 (8)	14 (9.9)	7 (11.5)	1 (1.4)	
Response					0.11
MPR	148 (54)	70 (49.3)	30 (49.2)	48 (67.4)	
pCR	82 (29.9)	40 (28.1)	17 (27.9)	25 (35.2)	
Non-MPR	126 (46)	72 (50.7)	31 (50.8)	23 (32.4)	

ADC, adenocarcinoma; MPR, major pathological response; pCR, pathological complete response; SCC, squamous cell carcinoma; SD, standard deviation.

a Comparisons were conducted between the internal cohort and external cohort.

Moreover, as exhibited in Table 2, the external validation cohort was comprised of 15 patients from Ningbo Hwa Mei Hospital, 11 patients from The First Affiliated Hospital of Nanchang University and 45 patients from Sir Run Run Shaw Hospital. Patients in The First Affiliated Hospital of Nanchang University in an earlier pretreatment stage (T1 stage: n = 5, 45.5%, P = 0.015; N0 stage: n = 9, 81.8%, P = 0.010; stage I: n = 7, 63.6%, P < 0.001), had a higher proportion of lobectomy (n = 11, 100%, P = 0.008), and had an earlier pathological N stage (N0: n = 9, 81.8%, P = 0.029).

**Table 2:** Clinicopathological characteristics of patients in the external validation cohort.

Characteristics	NB cohort (n = 15)	NC cohort (n = 11)	SRRS cohort (n = 45)	P value^a^
Age, mean ± SD, years	65.2 ± 7.2	64.7 ± 8.4	62.9 ± 6.8	0.56
< 65	8 (53.3)	6 (54.5)	23 (51.1)	
≥65	7 (46.7)	5 (45.5)	22 (48.9)	
Gender				0.70
Female	1 (6.7)	0	2 (4.4)	
Male	14 (93.3)	11 (100)	43 (95.6)	
Smoking status				0.24
Never smoked	7 (46.7)	3 (27.3)	25 (55.6)	
Smoker	8 (53.3)	8 (53.3)	20 (44.4)	
Pretreatment T stage				0.02
T1	1 (7.1)	5 (45.5)	4 (8.9)	
T2	6 (42.9)	6 (54.5)	17 (37.8)	
T3	4 (28.6)	0	16 (35.6)	
T4	3 (21.4)	0	8 (17.8)	
Pretreatment N stage				0.01
N0	7 (46.7)	9 (81.8)	11 (24.4)	
N1	3 (20)	1 (9.1)	10 (22.2)	
N2	5 (33.3)	1 (9.1)	24 (53.3)	
Pretreatment TNM stage				<0.001
I	1 (6.7)	7 (63.6)	3 (6.7)	
II	5 (33.3)	3 (27.3)	8 (17.8)	
III	9 (60)	1 (9.1)	34 (75.6)	
Surgical procedure				0.01
Lobectomy	9 (60)	11 (100)	37 (82.2)	
Bilobectomy	1 (6.7)	0	6 (13.3)	
Pneumonectomy	5 (33.3)	0	2 (4.4)	
Pathological N stage				0.03
N0	6 (40)	9 (81.8)	31 (68.9)	
N1	5 (33.3)	0	3 (6.7)	
N2	4 (26.7)	2 (18.2)	11 (24.4)	
N downstage in pretreatment N2 disease				0.03
N2 to N0	0	1 (100)	14 (58.3)	
N2 to N1	3 (60)	0	2 (8.3)	
N2	2 (40)	0	8 (33.3)	
Histology				0.38
SCC	11 (73.3)	11 (100)	33 (73.3)	
ADC	4 (26.7)	0	11 (24.4)	
Others	0	0	1 (2.2)	
Response				0.07
MPR	12 (80)	9 (81.8)	28 (60)	
pCR	3 (20)	6 (54.5)	16 (35.5)	
Non-MPR	3 (20)	2 (18.2)	18 (40)	

ADC, adenocarcinoma; MPR, major pathological response; NB, Ningbo Hwa Mei Hospital; NC, The First Affiliated Hospital of Nanchang University; pCR, pathological complete response; SCC, squamous cell carcinoma; SD, standard deviation; SRRS, Sir Run Run Shaw Hospital.

a Comparisons were conducted among the NB cohort, NC cohort and SRRS cohort.

### Predictive performance the deep learning model

**Fig. 2:**
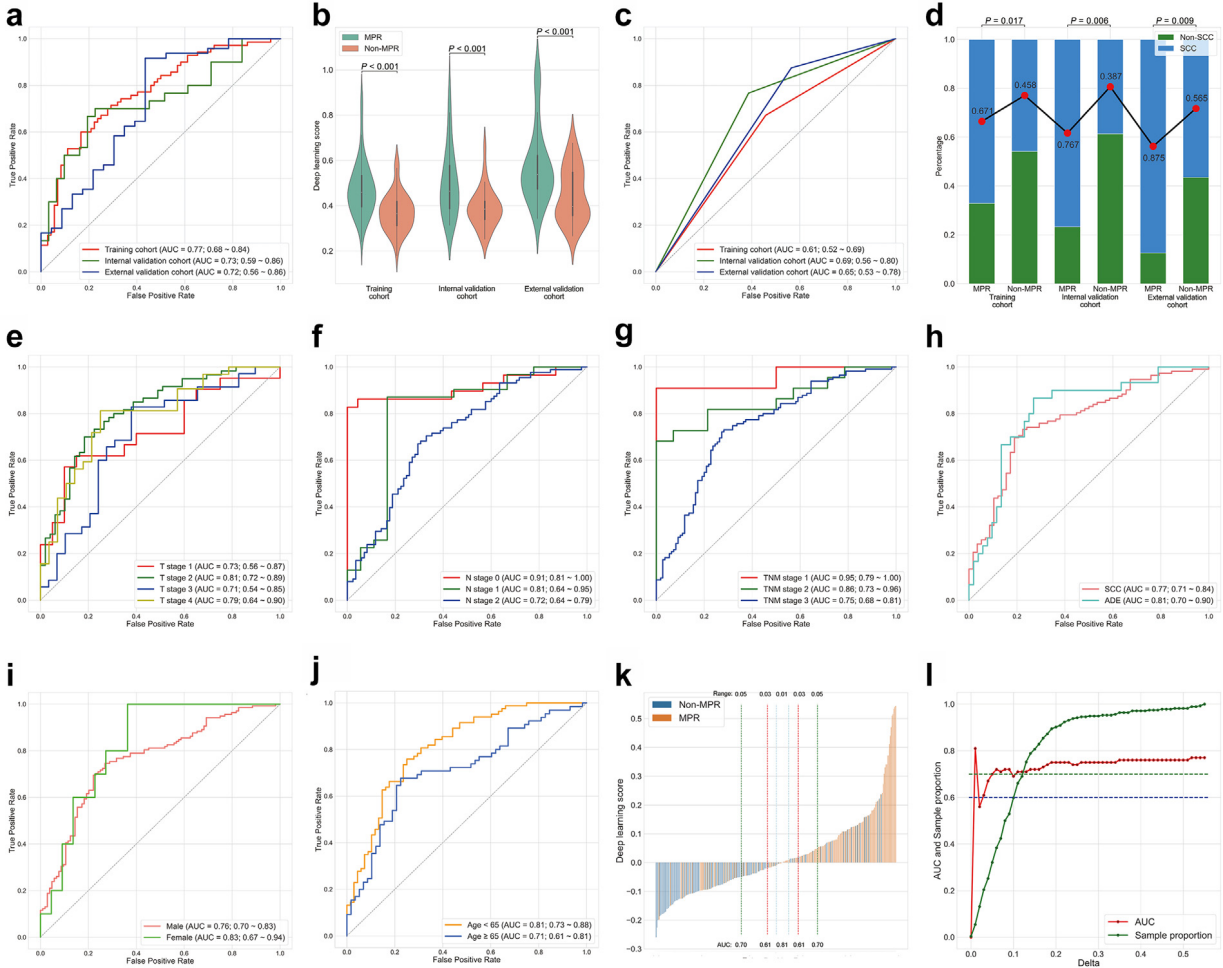
**The performance of the deep learning model and clinical model for predicting the MPR to neoadjuvant chemoimmunotherapy**. (a) ROC curves of the deep learning model in three cohorts; (b) The box figure shows the distribution of the deep learning score between MPR and non-MPR groups in three cohorts; (c) ROC curves of the clinical model in three cohorts; (d) Histograms shown the percentage of squamous cell carcinoma between MPR and non-MPR groups in three cohorts; (e–j) ROC curves of the deep learning model based on the clinicopathologic factors, including the pretreatment clinical T stage (e), N stage (f), TNM stage (g), Histologic subtype (h), gender (i) and age (j) in the whole population; (k) Waterfall plot for deep learning score in the whole population. (l) Line chart for delta range of deep learning score and AUCs in the whole population. AUC, area under the curve; MPR, major pathological response; ROC, receiver operating characteristic curve; SCC, squamous cell carcinoma.

The deep learning model based on Shufflenetv2x05 shows the optimal performance (Table S1). The AUC to distinguish MPR was 0.77 (95% confidence interval [CI]: 0.70–0.84), 0.73 (95% CI: 0.58–0.86) and 0.72 (95% CI: 0.58–0.85) in the training cohort, internal validation cohort and external validation cohort, respectively (Fig. 2a). The results of each step of the model training are recorded in Table S2.

**Fig. 3:**
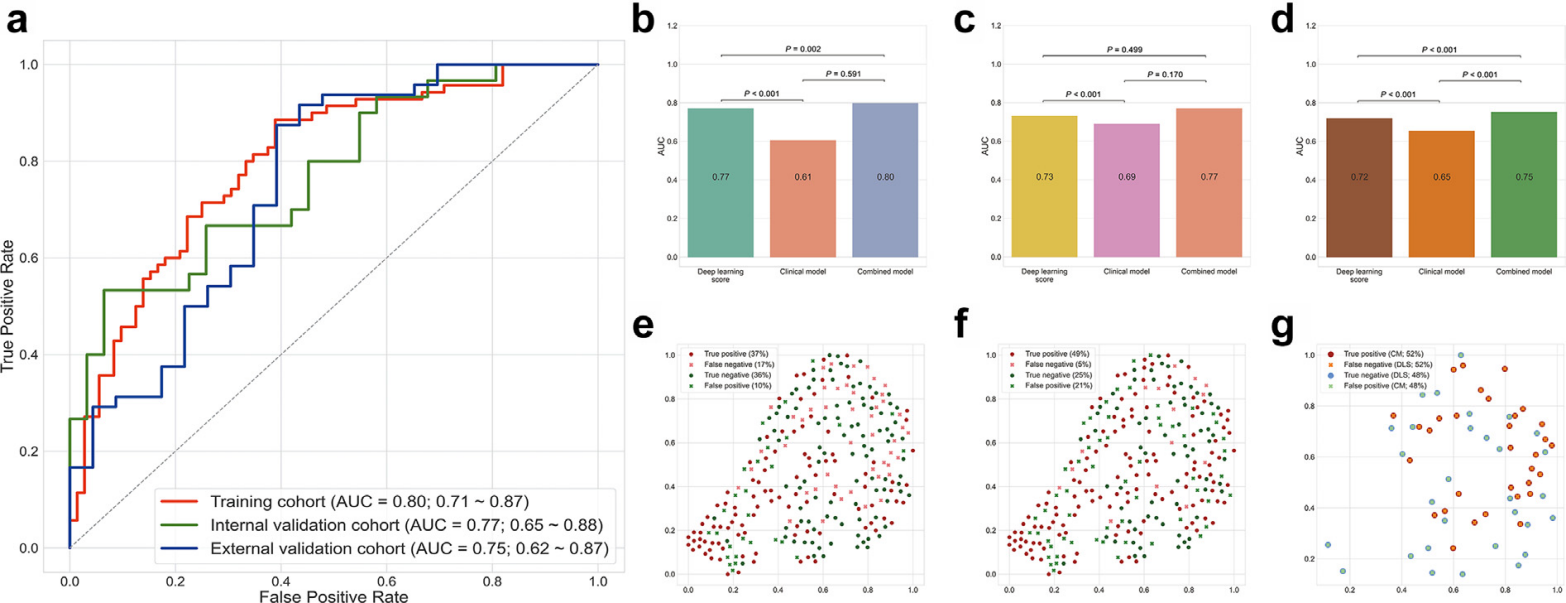
**The performance of the combined model for predicting the MPR to neoadjuvant chemoimmunotherapy**. (a) ROC curves of the combined model in three cohorts; (b) DeLong test for AUCs among the clinical model, deep learning model and combined model in the training cohort; (c) DeLong test for AUCs among the clinical model, deep learning model and combined model in the internal validation cohort; (d) DeLong test for AUCs among the clinical model, deep learning model and combined model in the external validation cohort; (e) Distribution graph for two-dimensional spatial structure and diagnostic metrics of the deep learning score in the whole population; (f) Distribution graph for two-dimensional spatial structure and diagnostic metrics of the combined score in the whole population; (g) Distribution graph for two-dimensional spatial structure and diagnostic metrics of the deep learning score and combined score for patients with different predicted outcomes between two models in the whole population. AUC, area under the curve; MPR, major pathological response; ROC, receiver operating characteristic curve.

Patients achieving MPR were associated with a significantly higher deep learning score than those with non-MPR in all three cohorts (Fig. 2b). In the construction of the clinical model, only the histological type of squamous cell carcinoma (odds ratio: 2.415; 95% CI: 1.222–4.771; P = 0.011) proved to be independently associated with a lower probability of MPR after the multivariable logistic regression-based backward selection (Table 3 and Fig. S1). Although the clinical model could screen out the patients with MPR to a certain extent, with AUCs of 0.61 (95% CI: 0.53–0.69), 0.61 (95% CI: 0.53–0.69) and 0.65 (95% CI: 0.53–0.77) in the training cohort, internal validation cohort and external validation cohort (Fig. 2c and d), respectively, its predictive efficiency was significantly poorer than the deep learning model (Fig. 3b–d).

**Table 3:** Univariable and multivariable analyses for major pathological response in the training cohort.

Variables	Univariable		Multivariable	
	OR (95% CI)	P value	OR (95% CI)	P value
Age (≥65)	1.114 (0.574–2.160)	0.75		
Gender (male)	2.807 (1.021–7.721)	0.04		
Smoking status (ever smoked)	1.179 (0.605–2.295)	0.63		
Maximam diameter	1.002 (0.992–1.011)	0.73		
Pretreatment T stage				
T1	Reference			
T2	1.871 (0.684–5.113)	0.22		
T3	2.100 (0.695–6.349)	0.19		
T4	1.625 (0.514–5.136)	0.41		
Pretreatment N stage				
N0	Reference			
N1	3.273 (0.883–12.125)	0.08		
N2	1.843 (0.587–5.788)	0.30		
Pretreatment TNM stage				
Ⅰ	Reference			
Ⅱ	1.143 (0.060–21.870)	0.93		
Ⅲ	0.953 (0.058–15.579)	0.97		
Histology (SCC)	2.415 (1.222–4.771)	0.01	2.415 (1.222–4.771)	0.01

CI, confidence interval; OR, odds ratio; SCC, squamous cell carcinoma.

We also established different subgroups by clinical characteristics to evaluate the performance stability of the deep learning model (Fig. 2e–l), indicating that the deep learning model achieved relatively stable and satisfactory predictive performance in certain subgroups with the clinical characteristics, such as TNM stage (stage Ⅰ: AUC = 0.95; stage Ⅱ: AUC = 0.86; stage Ⅲ: AUC = 0.75) (Fig. 2g), tumour pathological subtype (squamous cell carcinoma: AUC = 0.77; adenocarcinoma: AUC = 0.81) (Fig. 2h) and gender (male: AUC = 0.76; female: AUC = 0.83) (Fig. 2i). In the remaining subgroups, there were weak aspects of the deep learning model, such as the T3 subgroup (Fig. 2e; AUC: 0.71), N2 subgroup (Fig. 2f; AUC: 0.72) and older subgroup (Fig. 2j; AUC: 0.71).

To further evaluate the clinical utility of the deep learning model, we attempted to find the score intervals with better and worse predictions in the deep learning model. When a patient's score falls within the better prediction range, clinicians can give more trust to the model. Ultimately, we calculated the cutoff value of the deep learning score as 0.439 based on the Youden Index. The waterfall plot, which ranks patients' scores from smallest to largest, shows patients with a deep learning score higher than the cutoff value were mostly MPR (Fig. 2k). Subsequently, we gradually increased the calculation interval of AUC by delta (0.01 as a change unit) starting from the cutoff value of 0.439 (Fig. 2l). We found that patients with a deep learning score between 0.429 and 0.449 (delta = 0.01) had the highest AUC of 0.81, which then dropped sharply to 0.56 at a delta of 0.02, and stabilized above the AUC of 0.60 and 0.70 at deltas of 0.03 and 0.05, respectively. Hence, as the delta increases from 0.03, doctors can give the model more confidence in clinical use. When the delta is less than 0.03, doctors need to integrate other markers to make decisions. It should be noted that the delta is the absolute value of the difference between the score and the cut-off value.

### Multifactorial exploration of deep learning model and clinical characteristics

In the preceding results, we generated the image-based deep learning model and clinical model. To obtain the most accurate quantification for the probability of MPR to neoadjuvant chemoimmunotherapy, we fit the deep learning model and clinical model together by logistic regression to construct a combined model. The combined model achieved better performance than the deep learning model in the training cohort (Fig. 3a; AUC: 0.80, 95% CI: 0.72–0.87), the internal validation cohort (Fig. 3b; AUC: 0.77, 95% CI: 0.64–0.89), and the external validation cohort (Fig. 3a; AUC: 0.75, 95% CI: 0.62–0.87), and a significant difference was observed in the training cohort (P = 0.002) and the external validation cohort (P < 0.001), but not in the internal validation cohort (P = 0.499) (Fig. 3b–d).

In addition, we revealed the deep learning features output from the convolutional layers of the model, and plotted the predicted distributions of the deep learning score (Fig. 3e) and combined score (Fig. 3f) after reducing them to two dimensions using t-SNE.^22^ The distribution graphs of the two models both indicated that the deep learning features had a certain spatial structure difference in the two-dimensional space of t-SNE, and the introduction of the fully connected network enabled the model to construct a nonlinear discrimination surface based on the differences in the feature space. This result also reflected the need to construct high-dimensional hidden layers in the classification module. In addition, we extracted the samples using different discrimination between the combined score and deep learning score to draw a distribution graph (Fig. 3g), which clearly showed that the combined model increased the number of positive samples with higher accuracy than the deep learning model.

### Biological basis exploration

**Fig. 4:**
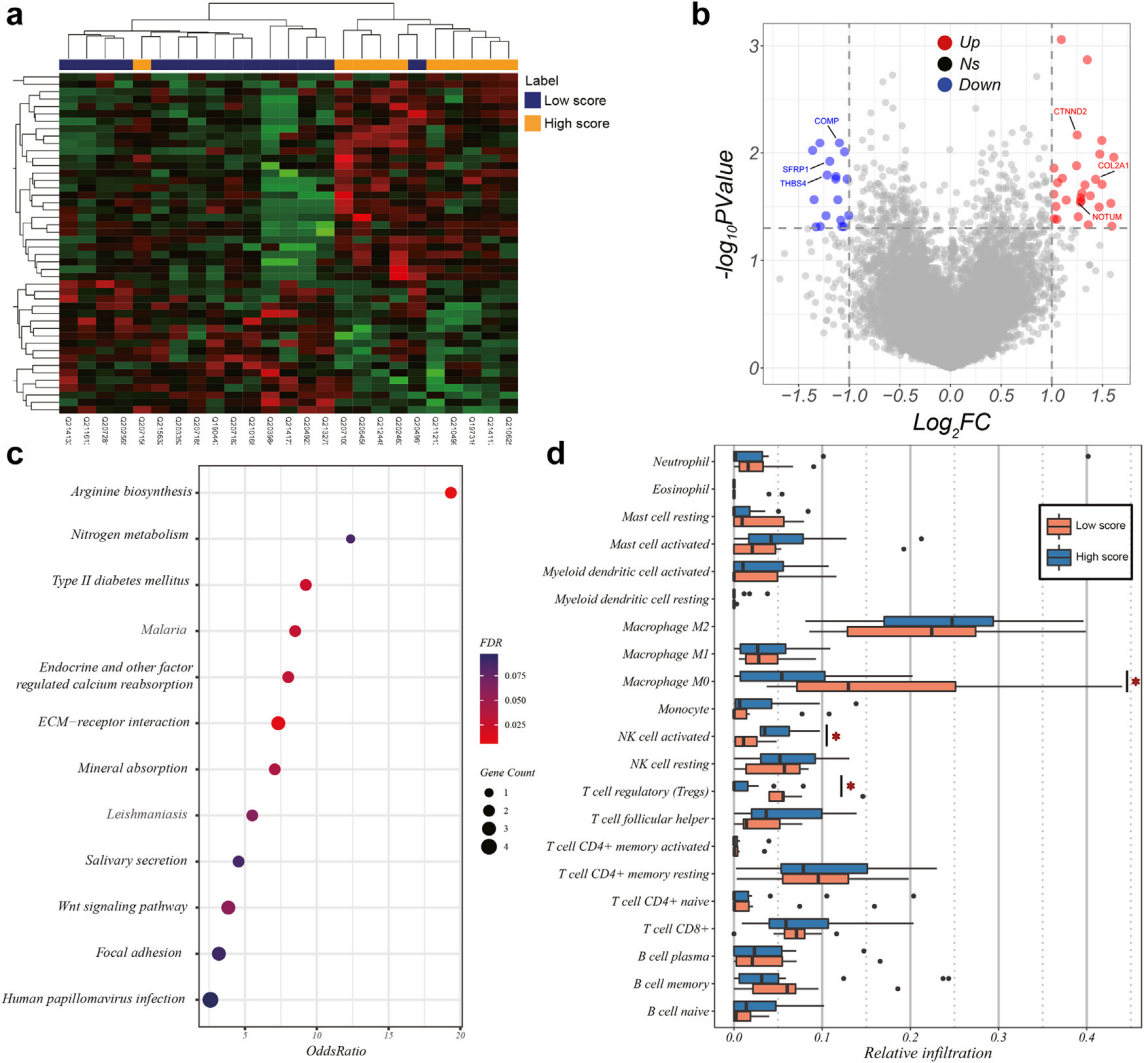
**The genetic analysis for investigating the underlying biological basis of the developed deep learning model**. (a) Heatmap of z-score normalized gene expressions presenting the differential expressed genes in samples categorized as low deep learning score compared with that categorized as high deep learning score; (b) Volcano diagram of gene expression profiles in samples separated by low deep learning score versus high deep learning score. The red dots represent genes upregulated in patients categorized as high score, whereas the blue dots represent genes upregulated in patients categorized as low score. The x-axis denotes the fold change (log2 scale), whereas the y-axis indicates statistical significance (−log10 format); (c) Bubble plot of the top 10 enriched pathways identified by gene enrichment analysis for the set of differential expressed genes, ranked by the odds ratio; (d) Box plot representing the estimation of the abundances of member cell types in a mixed cell population. FDR, false discovery rate. (For interpretation of the references to colour in this figure legend, the reader is referred to the Web version of this article.)

As illustrated in Fig. 4a and b, significant differences were proved regarding gene expression between 10 high-score patients and 15 low-score patients. The differential expressed genes were mainly distinguished as two clusters, which was in accordance with divisions based on the deep learning score. For tumours with a low deep learning score, pathways promoting tumour proliferation, such as ECM−receptor interaction (enriched genes: COL2A1, COMP, and THBS4), Wnt signaling pathway (enriched genes: CTNND2, SFRP1, and NOTUM) and focal adhesion (enriched genes: COL2A1, COMP, and THBS4), were significantly unregulated (Fig. 4c). In addition, tumours categorized as a high score exhibited less infiltrated M0 macrophage and regulatory T cells, but exhibited more activated NK cells than those categorized as a low score (Fig. 4d).

## Discussion

Neoadjuvant chemoimmunotherapy, which allows for conferring superiority in increasing the chance of radical resection and improving prognosis compared to conventional neoadjuvant chemotherapy, has been a promising treatment for NSCLC.^5^, ^6^, ^7^, ^8^ Despite the tremendous advancements, a significant proportion of NSCLC population could not achieve MPR to neoadjuvant chemoimmunotherapy.^9^ In such instances, an effective signature for screening out patients potentially benefiting from this state-of-the-art therapeutic strategy is urgently needed. The current multicentre study constructed a CT-based deep learning model to predict MPR probability in NSCLC after neoadjuvant chemoimmunotherapy. By integrating the deep learning score and clinical score, the combined model achieved AUCs of 0.77 and 0.75 in the internal validation and external validation cohorts, respectively.

The pathological response has served as a well-recognized surrogate in neoadjuvant immunotherapy trials for numerous solid tumours.^23^ Unlike other tumours such as breast and bladder cancers, the proportion of pCR in NSCLC post-chemoimmunotherapy was relatively scarce, only 9%–63%.^24^ In contrast, MPR could be achieved in 27%–86% of NSCLC patients after neoadjuvant chemoimmunotherapy,^24^ indicating that setting MPR as the predictive endpoint could make the training sample more balanced and recognize almost twice as many patients potentially benefiting from neoadjuvant chemoimmunotherapy compared to pCR. Recently, preliminary results of the NADIM study also revealed that MPR was associated with significantly improved 1-year progression free survival (88.4% versus 57.1%, P = 0.01) in NSCLC patients undergoing neoadjuvant chemoimmunotherapy,^7^ emphasizing the importance of predicting MPR. In addition, a potential concern for utilizing MPR is the lack of precision due to the inter and intra-observer variability in pathological evaluation. Weissferdt et al.^25^ conducted a prospective trial, where two pathologists independently quantified the residual tumour cells percentage in the resected specimens after neoadjuvant therapy. The diagnostic agreements between the pathologists were satisfactory (R^2^ = 0.994), further supporting the rationality of predicting MPR. Therefore, in this study, we selected MPR as the main predicting endpoint.

CT is a routine modality used in clinical practice to evaluate the post-therapy response in NSCLC. To a certain extent, lesion diameters in CT images could directly reflect tumour burden, and changes in diameter might provide dynamic monitoring for the therapeutic response. Evidence has emerged on the feasibility of tumour diameters in CT for predicting the MPR of neoadjuvant chemotherapy and target therapy. However, in the unique context of chemoimmunotherapy, where drugs mediately suppress the growth of tumour cells by leveraging the activation of the immune system, meaning the therapeutic response might have occurred prior to the regression of tumour gross size,^26^ radiological regression could not accurately imply pathological regression after neoadjuvant chemoimmunotherapy, which has been demonstrated in clinical trials.^7^ In certain case with MPR, the tumour radiological size might even become larger due to immune cell infiltration. Thus, superficial CT characteristics were incapable of accurately predicting the pathological response, and high dimensional and throughput imaging features should be extracted to quantify the probability of MPR after neoadjuvant chemoimmunotherapy in the NSCLC population.

The advent of deep-learning-based radiomics, which harbors the potential of mining the deep-level imaging features not being recognized by the human eye,^10^ shed new light on the prediction of neoadjuvant immunotherapeutic efficacy. The application of deep learning for the radiomics analysis of tumours has been playing an increasingly significant role in disease diagnoses, treatment decisions, and prognosis predictions.^11^, ^12^, ^13^ Numerous studies have confirmed that significant associations exist between CT radiomics features and immunotherapy response in advanced NSCLC receiving ICI treatment,^14^^,^^27^^,^^28^ which provided a rationale for conducing the current study. Furthermore, in the context of neoadjuvant therapy, previous publications have managed to construct radiomics models to obtain the pretest probability of pathological responses after neoadjuvant chemotherapy in various tumours including NSCLC, achieving AUCs of 0.63–0.73.^29^^,^^30^ Despite all these efforts, no study has attempted to investigate the feasibility of radiomics representations in predicting neoadjuvant immunotherapeutic efficacy.

Our study resorted to the deep learning algorithm to generate a CT radiomics model for quantifying the MPR probability in NSCLC receiving neoadjuvant chemoimmunotherapy, and after integrating the clinical characteristics, the combined model achieved an AUC of 0.75 in the multicentre external validation cohort. Although the efficiency was not adequate to serve as a direct determinant of MPR, the proposed model could assist doctors in optimizing the administration of neoadjuvant chemoimmunotherapy for NSCLC. On the one hand, for patients predicted to have a high probability of MPR, further molecular tests could be conducted to further evaluate the suitability for neoadjuvant immunotherapy. On the other hand, in patients predicted to have a low possibility of MPR, invasive core biopsies and expensive molecular tests might be avoided, which hinted at its utility in potentially recognizing NSCLC patients who might be sensitive to neoadjuvant chemoimmunotherapy.

The main drawback of the deep learning model is its inability to be interpreted, which posed a stubborn conundrum on the deployment of this black-box technique in clinical practice. Helping doctors understand these nameless features and the underlying mechanism of their predictive ability requires further elucidation. To study the prediction process of our proposed model, we output the areas deemed important by the network through the visualization method (Supplement), it was worth mentioning that the tumour microenvironment played an irreplaceable role in predicting neoadjuvant immunotherapeutic efficacy, which was supported by previous studies.^14^^,^^28^ To move forward, we adopted gene analyses of the RNA-sequencing data to uncover the biological basis of the deep learning model, finding that the deep learning phenotypes were associated with the pathway of tumour proliferation, which in turn supported the fact that high deep learning score was associated with a greater likelihood of MPR.

Limitations still exist regarding the current study. First, due to the retrospective nature of the study, patient selection bias and potential deviations in the MPR distribution were inevitable. Future large-scale studies with multiethnic patients in a prospective design are still warranted. Second, all patients included were treated after 2019; thus, is remains unclear the deep learning features or related infiltrative component are associated with survival outcomes. Future studies should include the endpoint of survival to comprehensively evaluate the predictive efficiency of our proposed model. Finally, only CT modality was adopted in the model construction, so room for improvement remains in terms of the precision of the algorithm. In subsequent studies, we will increase the modalities input into the network to optimize the accuracy of the deep learning for predicting neoadjuvant immunotherapeutic efficacy.

## Conclusions

This is the first study to investigate the predictive value of deep learning features for neoadjuvant chemoimmunotherapeutic efficacy in NSCLC. The proposed deep learning model based on CT images can effectively predict MPR in NSCLC patients treated with neoadjuvant chemoimmunotherapy. Moreover, the underlying biological basis of deep learning score may be related to the pathways mediating tumour proliferation and the promotion of antitumour immune cell infiltration in the microenvironment.

## Contributors

Y.S., B.H., F.W. and Y.Z. designed this study and wrote the paper. Y.S., B.H., F.W. and Y.Z. built the deep learning models. T.W. and Z.L. processed and analysed the data. M.Y., B.Y., J.D., X.S., C.W., L.H., Y.Z. and Y.Y. collected the clinical dataset and performed data preprocessing. H.H., D.D., C.C. and J.T. conceived the project and edited the paper. All authors reviewed and approved the final manuscript for submission. We ensured that all authors had access to all the raw datasets. Y.S., B.H., F.W. and Y.Z. have verified the data and are independent of any company or investor. J.T. had full access to all the data in the study and had final responsibility for the decision to submit for publication.

## Data sharing statement

The CT imaging data and clinical information in the current study are not publicly available for patient privacy purposes but are available from the corresponding authors upon reasonable request. The proposed source codes are provided at GitHub (https://github.com/Bercy0616/Prediction_of_efficacy_of_Neoadjuvant_Immunotherapy).

## Declaration of interests

We declare no competing interests.

## Appendix A Supplementary data

Supplementary Material

Caption for Supplementary Material
