# Supplementary material for: Deep learning for predicting major pathological response to neoadjuvant chemoimmunotherapy in non-small cell lung cancer: A multicentre study
Source: eBioMedicine. 2022 Nov 14;86:104364. doi: 10.1016/j.ebiom.2022.104364 (PMC9672965; doi:10.1016/j.ebiom.2022.104364)

**Supplement**

**Packages**

When we used Python for analysis, four packages named "Stats", "Pytorch", "Scikit-Learn" and "Matplotlib" were included. "Stats" was used to acquire the test results; "Pytorch" (version 1.10.1; <https://pytorch.org/)> was used to build the deep learning network; "scikit-learn" (version 0.21.3; https://scikit-learn.org/stable/) was used to calculate the quantitative metrics of the model, such as AUC; "matplotlib" (version 2.2.2; <https://matplotlib.org/index.html>) was used to draw ROC and other curves.

When we used R for analysis, three packages named "rms", "GGPlot2", "GGRadar" “limma” and “clusterProfiler” were included. "rms" was used to carry out the logistic regression algorithm; "GGPlot2" and "GGRadar" were used to draw the radar plots; “limma” and “clusterProfiler” were adopted for genetic analyses.

**Visual analysis**

The algorithm to provide CT image feedback through the activation image is a crucial link in the research on artificial intelligence medical images. It allows the researcher to see the machine understand and judge the focus area of the sample; it is also expected to be accepted by clinicians in routine practice. In this study, we showed the category activation map by entering the bounding box of tumor images, category, and gradient-based Grad-cam algorithm to display the network (1). In addition, part of the convolutional kernel generated by the progressive judgment of the convolutional network was derived and displayed. The category activation map generated by Grad-cam can generate the focus areas for model discrimination. In addition, the feature map output by the convolution kernel shows the decision-making process of the model, thereby realizing the visualization of the whole process of the model.

To expound the prediction process of the deep learning model, we selected four patients in two categories to visualize the class-by-step feature maps and class activation map. As illustrated in Figure S2, the partial convolution kernel was concerned about both the lesion area and the peritumour area. After experiencing Stage 2 (one of the modules of Shufflenetv2), the feature maps gradually lost human eye observation capabilities. For the deep learning score, these features were combined with global information. After Stages 3 to 5, a total of 1024 feature maps were retained. The deep learning model used these feature maps of the tumour and peritumour area, and judged the efficacy after the fully connected network. In the class activation map, we could clearly observe that the imaging area of the tumor concentrated by the model was determined by the status of MPR or non-MPR.

**Reference**

1. Selvaraju RR, Cogswell M, Das A, Vedantam R, Parikh D, Batra D, editors. Grad-cam: Visual explanations from deep networks via gradient-based localization. Proceedings of the IEEE international conference on computer vision; 2017.

**Table S1:** Modeling results using different convolutional neural networks

|  | The AUCs of each cohort | | |
| --- | --- | --- | --- |
| Network name | Training cohort | Internal validation | External validation |
| Resnet18-3D | 0·74  (95%CI: 0·65-0·83) | 0·68  (95%CI: 0·53-0·80) | 0·66  (95%CI: 0·51-0·81) |
| Densenet121-3D | 0·76  (95%CI: 0·67-0·84) | 0·68  (95%CI: 0·54-0·81) | 0·70  (95%CI: 0·53-0·84) |
| Mobilenetv3-3D | 0·74  (95%CI: 0·65-0·82) | 0·70  (95%CI: 0·56-0·85) | 0·71  (95%CI: 0·55-0·86) |
| Shufflenetv2x05-3D | 0·77  (95%CI: 0·68-0·84) | 0·73  (95%CI: 0·59-0·86) | 0·72  (95%CI: 0·56-0·86) |
| Shufflenetv2x10-3D | 0·76  (95%CI: 0·66-0·83) | 0·71  (95%CI: 0·58-0·85) | 0·71  (95%CI: 0·55-0·85) |

AUC, area under the curve; CI, confidence interval.

**Table S2:** Stepwise results during model training

|  | The AUCs of each cohort | | |
| --- | --- | --- | --- |
| Network structure | Training cohort | Internal validation | External validation |
| Shufflenetv2x05-3D  +linear classifier | 0·73  (95%CI: 0·65-0·81) | 0·67  (95%CI: 0·53-0·80) | 0·68  (95%CI: 0·53-0·81) |
| Shufflenetv2x05-3D (frozen)  + fully connected network | 0·76  (95%CI: 0·68-0·83) | 0·71  (95%CI: 0·58-0·84) | 0·70  (95%CI: 0·55-0·84) |
| Shufflenetv2x05-3D  + fully connected network | 0·77  (95%CI: 0·68-0·84) | 0·73  (95%CI: 0·59-0·86) | 0·72  (95%CI: 0·56-0·86) |

AUC, area under the curve; CI, confidence interval.

**Figure S1:** Heat map for multivariable logistic analyses for major pathological response in the training cohort. SCC, squamous cell carcinoma; ADE, adenocarcinoma.


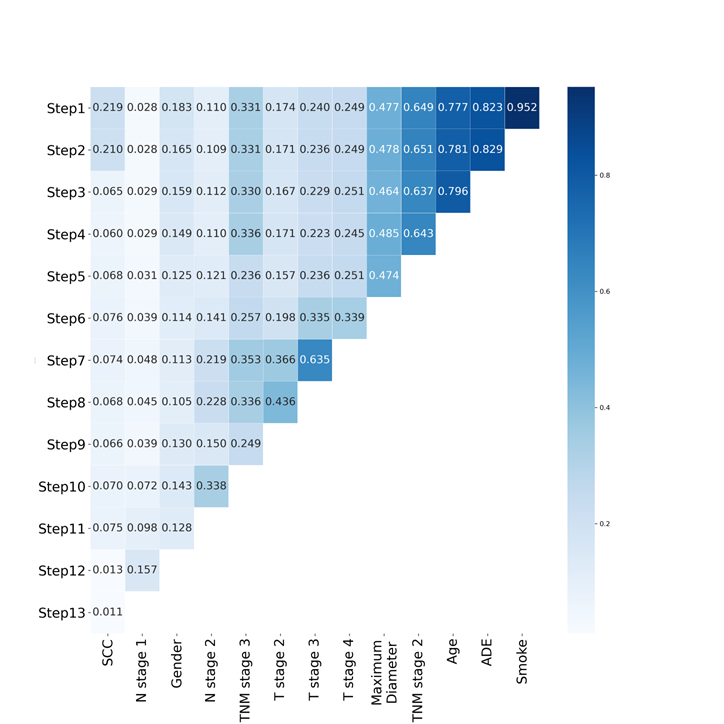


**Figure S2: The Visual analysis of deep learning model.** (Ⅰ) Four example cases with different baseline information and pathological response status; (Ⅱ) Feature maps in the network extracted from CT images; (Ⅲ) Class activation maps of four samples for two types of pathological response. MPR, major pathologic response; SCC, squamous cell carcinoma; ADE, adenocarcinoma.


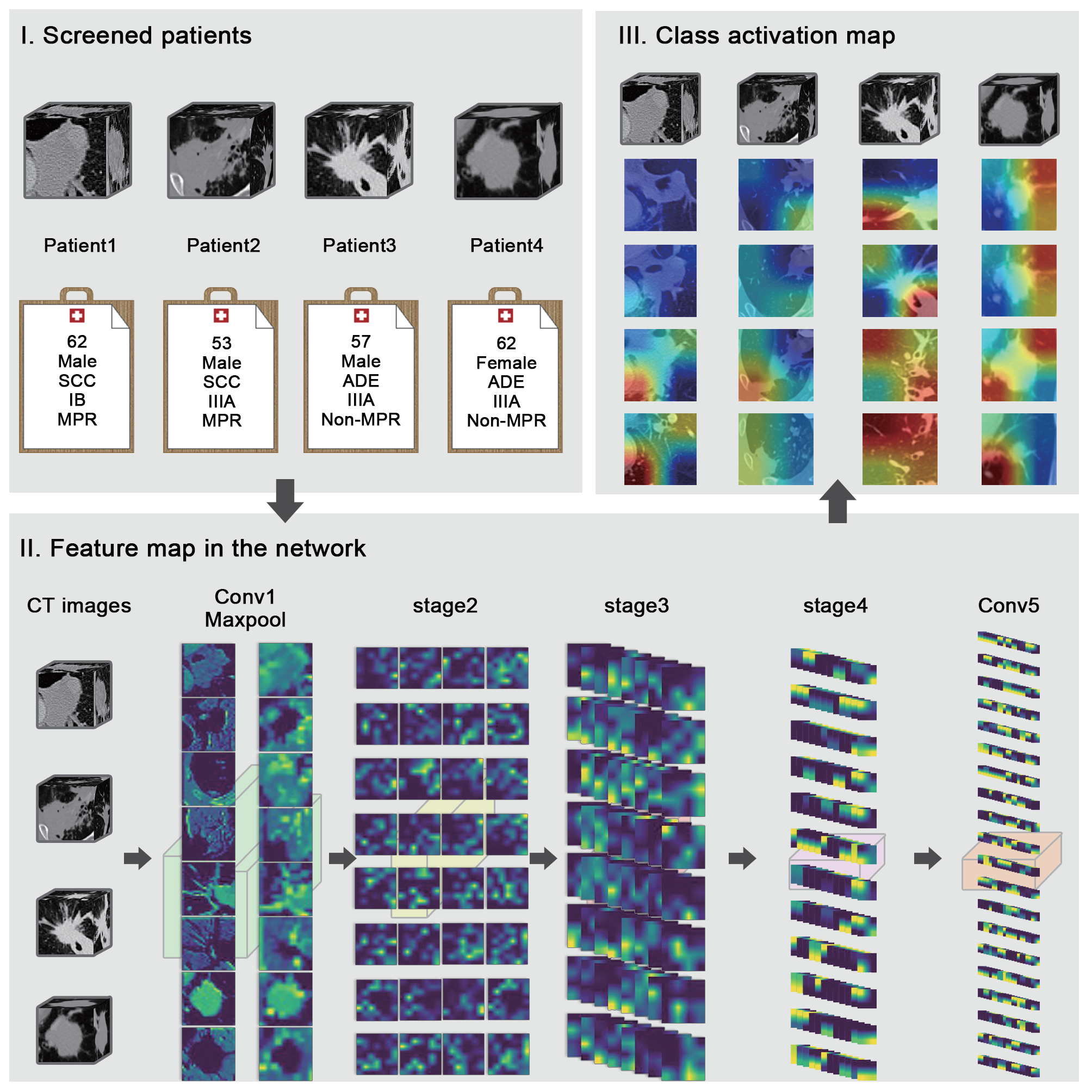

Supplement: Supplementary Material [file mmc1.docx]
