# Supplementary material for: Deep learning for predicting major pathological response to neoadjuvant chemoimmunotherapy in non-small cell lung cancer: A multicentre study
Source: eBioMedicine. 2022 Nov 14;86:104364. doi: 10.1016/j.ebiom.2022.104364 (PMC9672965; doi:10.1016/j.ebiom.2022.104364)
Supplement: Caption for Supplementary Material [file mmc2.docx]

1. Packages

2. Visual analysis

2. Table S1

3. Table S2

4. Figure S1

5. Figure S2
